# Impact of Contaminants on Microbiota: Linking the Gut–Brain Axis with Neurotoxicity

**DOI:** 10.3390/ijerph19031368

**Published:** 2022-01-26

**Authors:** Jordina Balaguer-Trias, Deepika Deepika, Marta Schuhmacher, Vikas Kumar

**Affiliations:** 1 Environmental Engineering Laboratory, Department of Chemical Engineering, Universitat Rovira i Virgili, 43007 Tarragona, Spain; jordina.balaguer@urv.cat (J.B.-T.); deepika@urv.cat (D.D.); marta.schuhmacher@urv.cat (M.S.); 2 IISPV (Pere Virgili Institute for Health Research), Sant Joan University Hospital, Universitat Rovira i Virgili, 43204 Reus, Spain

**Keywords:** in-vivo, in-silico, neurotoxicity, gut microbiota, BPA, DEHP, Chlorpyrifos, PFAS

## Abstract

Over the last years, research has focused on microbiota to establish a missing link between neuronal health and intestine imbalance. Many studies have considered microbiota as critical regulators of the gut–brain axis. The crosstalk between microbiota and the central nervous system is mainly explained through three different pathways: the neural, endocrine, and immune pathways, intricately interconnected with each other. In day-to-day life, human beings are exposed to a wide variety of contaminants that affect our intestinal microbiota and alter the bidirectional communication between the gut and brain, causing neuronal disorders. The interplay between xenobiotics, microbiota and neurotoxicity is still not fully explored, especially for susceptible populations such as pregnant women, neonates, and developing children. Precisely, early exposure to contaminants can trigger neurodevelopmental toxicity and long-term diseases. There is growing but limited research on the specific mechanisms of the microbiota–gut–brain axis (MGBA), making it challenging to understand the effect of environmental pollutants. In this review, we discuss the biological interplay between microbiota–gut–brain and analyse the role of endocrine-disrupting chemicals: Bisphenol A (BPA), Chlorpyrifos (CPF), Diethylhexyl phthalate (DEHP), and Per- and polyfluoroalkyl substances (PFAS) in MGBA perturbations and subsequent neurotoxicity. The complexity of the MGBA and the changing nature of the gut microbiota pose significant challenges for future research. However, emerging in-silico models able to analyse and interpret meta-omics data are a promising option for understanding the processes in this axis and can help prevent neurotoxicity.

## 1. Introduction

Human gut microbiota consists of about 10^14^ microbes with more than 1500 species, containing 100 times more genes than humans [1]. This extensive community of microorganisms is constituted mainly of bacteria and fungi, yeasts, viruses, and bacteriophages. From studies based on gene sequencing, the two main bacterial phyla that cover the human intestine are *Bacteroidetes* and *Firmicutes*, accounting for 90% of intestinal bacteria in healthy individuals. The remaining 10% are made up of *Proteobacteria, Actinobacteria, Verrucomicrobia,* and *Fusobacteria,* among others [2]. The distribution of the gut microbiota varies significantly between individuals and even changes at various stages in life [3]. The coevolution of the human being together with its microbiota has occurred due to a symbiotic relationship and co-dependency for the survival of both species, creating biomolecular networks between them [4].

Recent research has indicated that microbiota plays a crucial role in intestinal homeostasis and, consequently, host health. Genetics, environment, and lifestyle affect the composition of these intestinal microorganisms, which causes high variability even between people from the same region or who belong to the same family [5]. An increasing body of evidence points towards environmental factors playing a critical role in altering the gut-brain axis [6,7]. Daily exposure to industrial contaminants found in everyday products triggers changes in gut microbiota architecture. Gut dysbiosis, which refers to an imbalance of the microbial population and its metabolic capacity, seems to be the cause of the inflammation and disturbance of gut permeability [8]. Gut dysbiosis determines the number of molecules produced by the microbiota (metabolites, neurotransmitters, and fatty acids) that can penetrate the mucus and epithelial layers, hence affecting neuronal function and development and causing perturbation of the blood-brain barrier (BBB) [9]. The alteration of the bacterial metabolites and the weakening of tight junctions caused by the xenobiotics can allow the translocation of bacterial endotoxins in the bloodstream [10]. Microbiota is also in charge of promoting the digestion and absorption of nutrients that reach the intestine, neutralizing carcinogens and drugs, synthesizing vitamins (riboflavin and folate, B2 and B9, respectively), and helping the immune system mature. Therefore, intestinal microbiota composition is also affected by the type of diet and is directly related to the well-being of the host [11].

**Figure 1:**
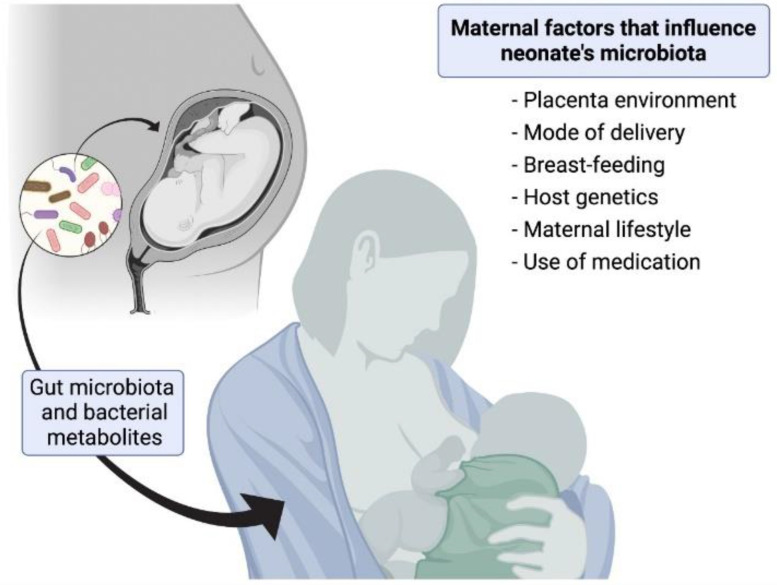
Maternal factors that influence neonate’s microbiota.

Furthermore, exposure to contaminants through daily food intake can generate toxicity and trigger neuroinflammation, promoting long-term effects [12]. A summary of maternal factors that influence neonate’s microbiota is presented in Figure 1. Among the different pollutants, scientific evidence points towards endocrine disruptor chemicals (EDCs), which can act like human hormones, alter the endocrine system, and be metabolized by the microbiota leading to dysbiosis and further health effects [13]. Some examples of EDCs are BPA, CPF, DEHP, and PFAS, which have been discussed in detail in later sections. Exposure to the EDCs in the developmental stage has been linked with several health effects like neurological diseases at different stages of life [14]. During the critical windows of development, elimination mechanisms, metabolism, and internal protective barriers (such as intestinal or BBB) are still underdeveloped, making infants more vulnerable to these toxic substances [15]. 

In order to understand the interplay between the gut microbiota, xenobiotics, and the brain, in-silico models—that integrate the information obtained from epidemiological, in-vivo and in-vitro studies—are required. In-silico models can simulate the interactions between the microbiome and the host, giving a mechanistic perspective of them [16]. According to the European Food Safety Authority (EFSA), the design of these approaches (machine-learning algorithms and computer simulations) is key to assessing chemical risk and its impact on the microbiota [17].

This review aims to understand the different pathways that relate microbiota and the brain, illustrate the latest research on the effect of these pollutants on microbiota together with early exposure role on the microbiota dysbiosis and developmental neurotoxicity. For this review, relevant in-vitro and in-vivo studies that address the issue related to microbiota and neurotoxicity were included. In addition to this, we highlighted improvements of the in-silico models and how they can emerge as a bridging tool between different experimental studies to improve risk assessment in humans.

## 2. Crosstalk between Microbiota and Nervous System: Gut-Brain Axis

The microbiota–intestine–brain axis is formed by the central nervous system (CNS), the autonomic nervous system (ANS), and the enteric nervous system (ENS) together with the neuroendocrine, neuroimmune, and obviously the microbiota.

The CNS consists of the brain and the spinal cord, and its primary function is to be the centre of processing. The ANS is divided into sympathetic (SNS) and parasympathetic (PNS) and is responsible for controlling the glands and internal organs. Lastly, the ENS directly controls the gastrointestinal system (motility, blood flow, and muscle secretion) and comprises two ganglionated neuronal plexuses [18]. The set of all these systems creates a neurohumoral communication network that also involves the microbiota in a bidirectional way, resulting in a complex interaction between the gut and the brain [19].

The communication pathways between the CNS and the microbiota include vagus nerve stimulation of the ENS signalling by neurotransmitters, hormones, or microbial metabolites, such as short-chain fatty acids (SCFA) or lipopolysaccharide (LPS). In addition, SCFA can be transferred from the intestine to the brain through the bloodstream crossing the BBB [20]. Animal studies have shown that the imbalance of the gut microbiota is capable of modulating brain chemistry, metabolic state, and neuronal function [21,22,23,24]. In order to better understand how this occurs, we have addressed this interaction from its three main pathways: neural, endocrine and immune.

### 2.1. Neural Pathway: Vagus Nerve and Neurotransmitters

**Figure 2:**
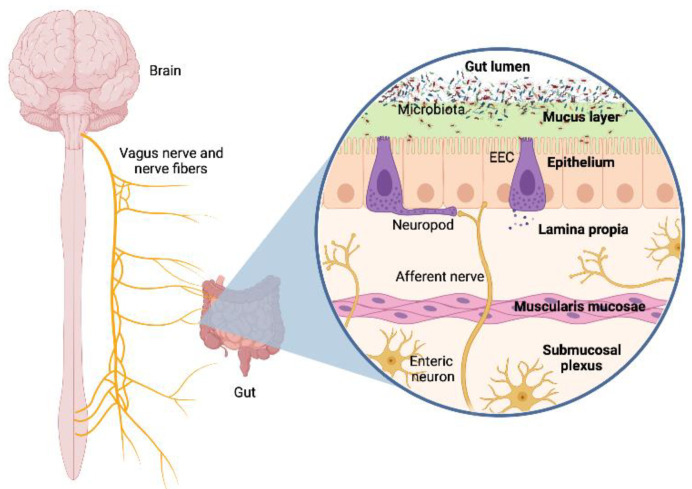
Scheme of the interaction between the microbiota and the vagus nerve.

The ENS is embedded in the walls of the intestine, regulating the essential gastrointestinal functions, but the spinal and vagal afferents hold control of the homeostatic state. The vagus nerve is the main component of the parasympathetic nervous system and a key piece in the gut–brain connection. Animal studies have shown that vagal integrity is crucial for communication between the intestine and brain [25]. The contact between the vagus nerve and the gut luminal microbiota is not direct because its afferent fibres do not cross the epithelial layer. Still, they can sense specific signalling of the microbiota or the enteroendocrine cells (EECs). These cells recognize microbial products through Toll-like receptors (TLR) [26]. Additionally, neuropod cells can synapse with neuronal circuits maintaining the communication with the plexuses of the ENS and consequently with the vagus nerve [27,28]. As the vagus nerve is the main afferent pathway, sensory input travels through the ganglia from the intestine to the brain. Through this mechanism, the intestinal microbiota participates or even regulates information transfer in this axis, as shown in Figure 2. 

The gut microorganisms secrete neuroactive substances; some of these are inhibitory neurotransmitters such as serotonin or 5-HT (*Enterococcus and Streptococcus*), y-aminobutyric acid (GABA) (*Bifidobacterium and Lactobacillus*), and glycine (*Bacillus*), and some are excitators such as dopamine (*Bacillus* and *Escherichia),* glutamate, and acetylcholine (ACh) (*Lactobacillus*) [29]. These substances can act directly on the ENS or indirectly through the EECs as described by Sarkar et al. [30]. In the case of SCFA, they activate the chemoreceptors of the vagal afferent fibres and can also cross the BBB, reaching the brain. In addition, SCFAs can regulate other neurotransmitters and enzymes to maintain the integrity of the BBB, for example, inhibiting the activity of histone deacetylase (HDAC) and the nuclear factor-kB. *Faecalibacterium prausnitzii* and *Roseburia* are the main SCFAs-producing bacteria; specifically, they are producers of butyrate. The decrease of these strains in the intestine is related to diseases such as Parkinson’s, Crohn‘s, or ulcerative colitis [31,32]. This phenomenon is partly explained by the role of butyrate as an energy source for colonic epithelium cells, in addition to being a stabilizer of the epithelial barrier function and having an anti-inflammatory effect on the mucosa [33]. Additionally, it has been observed that NaB (sodium salt of butyrate) has therapeutic effects on Huntington’s and Alzheimer’s disease models [34]. 

The microbiota also intervenes in the availability of the precursors and transporters of these neurotransmitters; for example, the production of tryptophan (a precursor of 5-HT) is regulated by metabolizing enzymes from the intestinal microbiota [35]. The results of several studies show that the microbiota can contribute to abnormal neurotransmitter metabolism, but there is still not enough information on the underlying mechanisms, so more research is needed in this field [36]. 

### 2.2. Endocrine Pathway: Hypothalamic-Pituitary-Adrenal Axis and Hormones

The microbiota also intervenes in the hypothalamic–pituitary–adrenal (HPA) axis, one of the central endocrine systems of the body, modulating the response to stress [37]. As described in the previous section, there can be a dysregulation of neurotransmitters and hence modification of the individual‘s hormone levels. This dysregulation affects the composition of the intestinal microbiota and influences the host‘s response. Another factor to consider is proinflammatory cytokines that can activate the HPA being produced by the immune system, which is in constant interaction with microbes [38]. In addition, the microbiota that inhabits the intestine functions as an endocrine organ by itself because it can produce and metabolize chemicals that are very similar to human hormones. In fact, bacterial products are capable of stimulating EECs and producing neuropeptides such as Y (NPY), YY, cholecystokinin, glucagon-like (GLP), and substance P, which acts as neurohormones [39].

One of the hormones that influence microorganisms are steroids, especially sex hormones. The levels of testosterone, estrogens, estradiol, and their metabolites have been shown to correlate with the diversity of the gut microbiota, leading to gender differences [40]. Specific bacterial genera such as *Clostridia* or *Ruminococcaceae* can transform estrogens, turning into bioavailable forms through the beta-glucuronidase secretion [41]. Another great example is cortisol, a glucocorticoid hormone released in response to stress, a process that promotes an increase in the permeability of the gastrointestinal tract. There is evidence of the association of cortisol production with increased *Firmicutes, Prevotella,* and *Enterobacteriaceae* [42] and decreased *Lachnospiraceae* and *Bifidobacterium* [43,44,45]. 

### 2.3. Immune Pathway: Microglia, Lymphocytes, and Cytokines

Between the brain and the intestine, there is a dynamic balance that the ENS and the HPA maintain, but a critical intermediary that interacts directly with these two is the immune system. The intestine is an immune organ itself as it provides a physical barrier and is highly populated with both adaptive and innate immune cells [46]. It acts as a defence against pathogens and separates them from the internal biological environment. To protect the intestine from excessive inflammation, the liver–brain–intestine neural arch has the role of an immunoregulatory niche and works as a feedback pathway [47].

The microbiota contributes to the maturation of the immune response; the microbial-associated molecular patterns (MAMPs) are the way it presents itself to the immune system. One of these is LPS, which activates or stimulates dendritic cells, macrophages, and neutrophils (in the case of the last, in combination with other stimuli) [48,49]. The activated innate system components produce inflammatory cytokines such as tumour necrosis factor-alpha (TNF-α) and interleukins 1 (IL-1) and 6 (IL-6). These cytokines can act on the afferent nerves and also cross the BBB, interacting with the microglia. The microglia are the glial cells that form the immune system of the CNS, being found mainly in the brain. Its primary function is to eliminate dead neurons and other cellular debris through phagocytosis and promote inflammation. The vagus nerve has been shown to activate the microglia, as this vagal signalling is necessary for the induction of T lymphocytes mediated by certain intestinal bacteria [50]. Another signalling pathway by which the microbiota stabilizes the microglia is SCFAs [51,52]. Both humans and rodents depend on bacteria to break down dietary fibre, and in this process of hydrolysis of the polysaccharides, SCFAs (butyrate, propionate or acetate) are obtained as products of fermentation. These SCFAs have the ability to inhibit cytokines such as IL-6 and IL-8 and therefore exert an anti-inflammatory action [53]. This evidence shows that the balance of the intestinal microorganisms plays a vital role in regulating the host‘s inflammatory response. It has already been commented that factors such as stress can condition this crosstalk and allow the passage of bacteria, their antigens, and endotoxins by increasing the permeability of the intestinal barrier. This, in fact, activates the immune system, causing a considerable increase in proinflammatory cytokines. These, together with 5-HT, influence corticotropin-releasing hormone (CRH), or arginine vasopressin (AVP), lead to altered cortisol and tryptophan availability [54]. Of the SCFAs, butyrate and propionate have been described as those having the most effects on the innate and adaptive immune system; this turns *Clostridia* spp. into a class of interest to study disorders such as inflammatory bowel diseases [55]. 

## 3. Contaminants, Microbiota and Neurotoxicity 

The importance of microbiota in human health has been widely described. However, it remains to be established to what extent EDCs exposure causes dysbiosis and affects human health.

The effect of environmental pollutants is a relevant field in public health. It is becoming increasingly clear that there is a toxic response from these compounds derived from industrial production, even in low concentrations. The intestinal microbiota is sensitive to changes in its environment and the habits of industrialized society seem to be the cause of its dysbiosis and progressive deterioration. In the last 100 years, there have been many changes: globalization, urbanization, and industrialization of the primary sector. All of them have had a significant impact on the lifestyle of the population and have resulted in the loss of ancestral organisms with critical functions in the individual [56]. Endocrine disruptors (EDs), heavy metals, pesticides, and other contaminants have effects on gut microorganisms, such as energy metabolism disorders. The side effects are related to the appearance of several of the diseases that prevail in developed countries [57,58]. We are talking about diabetes, obesity, liver diseases, infertility, asthma, neurodevelopmental problems, neurodegenerative diseases, and cancer [59]. Humans have the metabolic capacity to eliminate these lipophilic compounds that we recognize as foreign through a biotransformation process that consists of phases I, II, and III. In these, the different detoxification reactions take place. The gut microorganisms are also able to metabolize some of these xenobiotic compounds and transform them [60]. Reduction and hydrolysis are the mainly microbial enzymatic reactions (phase I), being β-glucuronidases, beta-glucosidases, azoreductase, nitroreductases, and aryl sulfatases its main enzyme types. In addition, there is evidence that the microbiota can regulate the expression of host genes related to this process [61].

**Figure 3:**
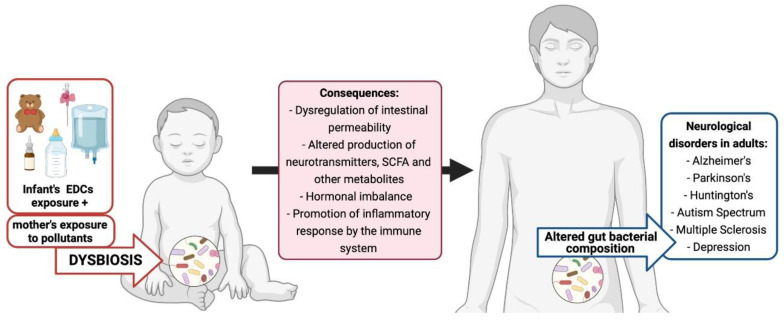
EDC exposure, early dysbiosis, and neurological disorders in adults.

That is why it is essential to consider the gut microbiota as one more parameter in evaluating toxicological risk [62]. Among all the aforementioned pollutants, the focus of this review is EDCs. A summary of early exposure, dysbiosis, altered biological pathways, and neurological disorders in adults is presented in Figure 3.

### 3.1. Endocrine Disruptors

EDs are exogenous chemicals or mixtures of them capable of mimicking human hormones and primarily interfere with the endocrine system [63]. EDs can bioaccumulate and have transgenerational effects [64]; this means that humans have a large body burden of them even if there is no pathological manifestation [65,66].

There has been continuous exposure to EDCs because they are being found in our everyday environment. Parabens, dioxins, furans, polychlorinated biphenyls, bisphenols, benzophenones, phthalates, flame retardants, and heavy metals are used for cosmetic products, bottles and plastic containers, cans, toys, pesticides and herbicides, electronic products, and even furniture [67,68,69,70]. Humans are continually exposed to EDCs through dermal, inhalation, or ingestion, with food being the major contributor [71]. EDCs can cross the placenta and are also found in breast milk, so they can induce developmental complications in infants [65]. The ability of EDCs to not follow dose-response patterns is the main limitation in studying their effects. Lower doses can have greater effects and, in addition, multiple exposures must be considered for the understanding effect of mixtures on human health [72]. 

#### 3.1.1. Bisphenol A 

One example of EDC is bisphenol A or BPA, an organic compound capable of binding to estrogen receptors. Exposure to it has been associated with diseases such as cancer, infertility, endometriosis, fatty liver disease, or obesity [67]. In humans as well as in other mammals, BPA is transformed through oxidation or hydroxylation reactions, but this degradation in the gastrointestinal tract is not complete, so BPA reaches the microbiota altering it significantly. Y. Wang et al. [73] used HepG2 cells with an in-vitro simulator of the human intestinal microbial ecosystem (SHIME) to show how parent BPA affects the microbial community. The results showed that *Lactobacilllus, Alcaligenes,* and *Mycobacterium* increased in the colon after exposure, suggesting that BPA exposure induces an increase in BPA-degrading bacteria and unifies the microbial community (More information in Table 1).

Microbial cultures isolated from samples obtained from the human intestine have made it possible to identify BPA tolerant strains (from 0.5 up to 50 ppm). Using next-generation sequencing (NGS), it was confirmed that *Firmicutes* are the main biodegradators of BPA. Within this phylum, *Bacillus* spp. stands out for having in its genome enzymes encoded by complete genes that catalyse the BPA degradation, mainly monooxygenases (P450) [74,75,76]. This family of enzymes in particular has relevant importance in the detoxification network and is the main one in phase I of the biotransformation [77].

In another in-vivo experiment by Feng et al. [78], BPA exposure at 50 μg/Kg BW/day (equal to tolerable daily intake (TDI) set by EFSA before January 2015) led to reduced microbial diversity and an increment of fat and lipids accumulation in the liver. *Proteobacteria* increased and *Verrucomicrobia* decreased within this phylum, specifically the *Akkermansia genus*, which is associated with a reduction in inflammation and an improvement in the function of the intestinal barrier. The results show that the intake of BPA at TDI dose damages the intestinal barrier and causes hepatic steatosis (NAFLD) and intestinal dysbiosis in mice. Furthermore, Lai et al. [79] compared the impact of a high fat diet (HFD) and a high sucrose diet (HSD) with the ingestion of BPA dissolved in water in male mice. The results showed that BPA exposure favoured the growth of *TM7* and *Proteobacteria*, especially *Helicobacteraceae*, but the populations of *Tenericutes* and *Firmicutes*, specifically of the *Clostridia* class, decreased (See in Table 1). 

A recent study has demonstrated that neurotoxicity caused by exposure to BPA in mice may be due in part to dysregulation of the microbiota–gut–brain axis [80]. The results of male mice showed that exposure to BPA from 0.5 mg/kg affected their cognitive abilities due to an increase in neuroinflammation. The levels of the neurotransmitter 5-hydroxytryptamine (5-HT) as well as its precursor tryptophan (TRP) and its metabolite 5-hydroxyindoleacetic acid (5-HIAA) decreased in serum, hippocampus, and colon. Furthermore, a decrease in Mucin 2 levels and mucus secretion in the colon was observed together with a reduction in propionic, caproic, and butyrate acid levels. The intestinal microbiota of these animals was notably altered. Due to BPA exposure, bacterial populations of the *Clostridia* and *Deltaproteobacteria* classes increased while *Bacteroidia, Erysipelotrichia, and Akkermansia* decreased significantly in male mice. In contrast to them, the cognitive function of females exposed was not significantly altered and unlike in males, the populations of *Bacteroidales* increased but *Betaproteobacteria* decreased (More details in Table 1). The Spearman analysis allowed us to identify the positive correlation of *Alloprevotella* and *Parabacteroidetes* with the expression of PSD-95 (related to the neuronal synapse), 5-HIAA, and the faecal levels of propionic and caproic acid. In contrast to the negative correlation of *Ruminiclostridium* with PSD-95, Occludin and Claudin-1 and acetic and caproic acids. Therefore, the data from this study show that the toxicity of BPA in animals is different depending on sex. In addition, the results show that BPA reduced the levels of faecal SCFA and 5-HT in the brain, as well as the abundance of bacteria related to TRP metabolism, which leads to an alteration in neurotransmitter signalling. BPA disrupted the intestinal barrier and the BBB integrity, which may be related to dysbiosis, thus promoting cognitive decline and inflammation in the colon and brain.

**Table 1:** Studies on exposure to BPA and its relationship with the intestinal microbiota.

BPA Exposure						
Cell Line/Species of Animal	Dose	Duration	Bacterial Genus	Bacterial Phylum	Impact *	Reference
HepG2 (Human)	25 μg/L, 250 μg/L and 2500 μg/L	10 days	*Lactobacilllus*	*Firmicutes*	↑	[73]
			*Alcaligenes*	*Proteobacteria*	↑	
			*Mycobacterium*	*Actinobacteria*	↑	
Male CD-1 mice	0.5 mg/kg of food	24 weeks		*Proteobacteria*	↑	[78]
			*Akkermansia*	*Verrucomicrobia*	↓	
			*Rikenella*	*Bacteroidetes*	↑	
Male CD-1 mice	20 mg/10 g body weight	10 weeks	*Helicobacter*	*Proteobacteria*	↑	[79]
				*TM7/Saccharibacteria*	↑	
			*Coprococcus*	*Firmicutes*	↓	
				*Firmicutes* (*Clostridia*)	↓	
			*Eubacterium*	*Firmicutes*	↓	
			*Lactobacillus*	*Firmicutes*	↓	
				*Tenericutes*	↓	
Male C57BL/6J	0.05, 0.5, 5 and 50 mg/kg/day (females only 50 mg/kg/day)	22 weeks	*Oscillibacter*	*Firmicutes*	↑	[80]
			*Tyzzerella*	*Firmicutes*	↑	
				*Firmicutes* (*Ruminococcaceae NK4A214 group*)	↑	
			*Alloprevotella*	*Bacteroidetes*	↓	
			*Ruminococcus*	*Firmicutes*	↓	
			*Parabacteroides*	*Bacteroidetes*	↓	
			*Akkermansia*	*Verrucomicrobia*	↓	
Male and female C57BL/6J			*Allobaculum*	*Firmicutes*	↓	
			*Muribaculum*	*Bacteroidetes*	↓	
			*Ruminiclostridium*	*Firmicutes*	↑	
			*Desulfovibrio*	*Proteobacteria*	↑	
Female C57BL/6J			*Bilophila*	*Proteobacteria*	↑	
			*Peptococcus*	*Firmicutes*	↑	
			*Bacteroides*	*Bacteroidetes*	↓	
			*Parasutterella*	*Proteobacteria*	↓	
			*Akkermansia*	*Verrucomicrobia*	↓	
			*Rikenella*	*Bacteroidetes*	↑	

Impact *: In this column, the increase (↑) or decrease (↓) refers to bacterial populations depending on the effect of the toxicity.

#### 3.1.2. Chlorpyrifos

Chlorpyrifos (CPF) is an organophosphate insecticide that irreversibly inhibits acetylcholinesterase (AChE), leading to a collapse in the nervous system of insects. Exposure to it can cause neurotoxic effects, reproductive, and developmental toxicity [81,82].

In an in-vitro study, Mendler et al. [83] investigated whether pesticides could affect the gut microbiota in the way they modulate the immune system (Mucosal-associated invariant T cells (MAIT cells)). They treated *Bifidobacterium adolescentis, Lactobacillus reuteri,* and *Escherichia coli* with CPF at 50–200 nM concentrations for 16 h. Of the three strains, *E. coli* showed a more significant response, and a negative correlation was detected between the concentration of CPF and known proteins of the folate biosynthesis pathway for *E. coli*. This leads to a dysregulation of the enzymes and an accumulation of the metabolites that activate the MAIT cells, causing an inflammatory response. 

Joly et al. [68] conducted an experiment with human cells and microbiota from healthy donors in SHIME, which showed that chronic exposure to low doses of CPF cause dysbiosis. Specifically, the proliferation of *Enterococcus* and *Bacteroides* and the decrease in *Lactobacillus* and *Bifidobacterium* in the colon reactors. In parallel, the same experiments were carried out on pregnant rats, giving similar results. Reygner et al. [84] repeated the study with almost the same results. Then, *Clostridia* also increased, and they concluded that chronic exposure to CPF could reduce SCFA and lactate production. Later, Réquilé et al. [85] also used SHIME together with another in-vitro model of the intestinal mucosa from Caco-2/TC7 cells to see the effect of CPF and the prebiotic inulin on them. They commented that the most severe dysbiosis was caused by the dose of 1 mg/day used in previous studies instead of by the highest concentration of 3.5 mg/day used in that study. They saw again that one of the microbial populations that decreased the most due to CPF was *Bifidobacterium,* followed by *Lactobacillus* (for more information on these data, see Table 2). 

On the other hand, J. W. Li et al. [86] focused on the relationship between dysbiosis produced by CPF and impaired endocrine function. The results showed that SCFA-producing bacteria and those related to testosterone were affected. *Streptococcus, Ruminiclostridium,* and *Coriobacteriaceae* increased significantly and *Romboutsia, Turicibacter*, and *Clostridium* decreased (Table 2). *Coriobacteriaceae* was associated with an increase in ghrelin and *Turicibacter* with the content of PYY. These results show how CPF affects the composition of the microbiota and its ability to induce hormone production due to its action as an endocrine disruptor in the HPA axis. As mentioned in Section 2.2, deregulation in the metabolism of certain hormones can cause alterations in brain function. 

**Table 2:** Studies on exposure to CPF and its relationship with the intestinal microbiota.

CPF Exposure						
Cell Line/Species of Animal	Dose	Duration	Bacterial Genus	Bacterial Phylum	Impact *	Reference
Pregnant females Hannover Wistar rat and their offspring and SHIME	1 mg/kg body weight/day	Gestation and 60 post-natal days	*Enterococcus*	*Firmicutes*	↑	[68]
			*Bacteroides*	*Bacteroidetes*	↑	
			*Lactobacilllus*	*Firmicutes*	↓	
			*Bifidobacterium*	*Actinobacteria*	↓	
Caco-2/TC7 cells And SHIME	3.5 mg/day	21–23 days post-seeding	*Lactobacilllus*	*Firmicutes*	↓	[85]
			*Bifidobacterium*	*Actinobacteria*	↓	
Male Wistar rats	0.3 mg/kg bw/day	20–25 weeks	*Streptococcus*	*Firmicutes*	↑	[86]
			*Ruminiclostridium*	*Firmicutes*	↑	
				*Actinobacteria*	↑	
			*Romboutsia*	*Firmicutes*	↓	
			*Turicibacter*	*Firmicutes*	↓	
			*Clostridium*	*Firmicutes*	↓	
			*Akkermansia*	*Verrucomicrobia*	↓	
			*Luteolibacter*	*Verrucomicrobia*	↓	
			*Prosthecobacter*	*Verrucomicrobia*	↓	
			*Coraliomargarita*	*Verrucomicrobia*	↓	

Impact *: In this column, the increase (↑) or decrease (↓) refers to bacterial populations depending on the effect of the toxicity.

#### 3.1.3. Diethylhexyl Phthalate

Diethylhexyl phthalate or DEHP is one of the most common phthalates and is mainly used as a plasticizer for the manufacture of PVC pieces or objects. The highest risk comes from the consumption of food or liquids that have come into contact with plastic [69,87]. Another wide use of DEHP is in medical devices, making plastics softer and more flexible. This fact is of concern because phthalate can reach the patient in higher concentrations and more directly, particularly in pregnant women and newborns [88,89]. The toxicity of DEPH comes mainly from its action as an androgen antagonist and is associated with obesity and insulin intolerance [90].

In-vivo studies showed that DEHP causes changes in the composition of the microbiota, decreasing its diversity and specifically altering the levels of bacterial metabolites. Fu et al. [91] observed that *Firmicutes*, *Turicibacter, Akkermansia*, *Verrucomicrobiales, and* *Romboutsia* increased a high dose whilst Bacteroidetes *Epsilonbacteraeota* and *Actinobacteria* decreased in all doses. Due to dysbiosis, the levels of SCFA, AA, and simple sugars were also significantly altered. On the other hand, G. Wang, Chen, et al. [92] found that the damage caused by DEHP varied depending on the rodent species (SD rats, Wistar rats, BALB/C mice, and C57BL/6J mice). SD rats underwent the most significant changes in their microbiota after being exposed, with a higher abundance of *Proteobacteria* and pathogenic bacteria such *Mycoplasma* or *Blautia* (associated with liver and intestinal diseases) and higher inflammatory response and decreased butyrate concentrations (more information in Table 3). This study suggests that the susceptibility to DEHP is related to the intestinal microbiota of each species.

**Figure 4:**
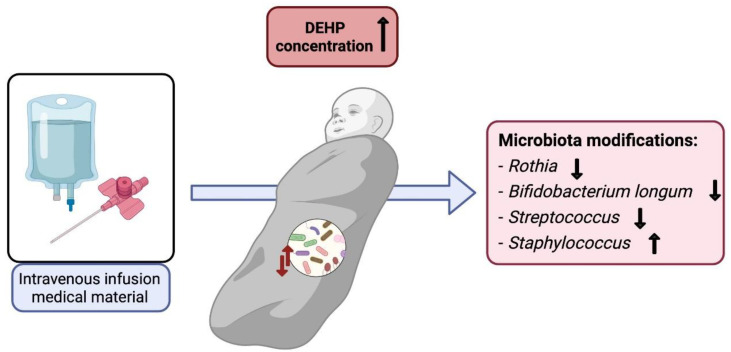
DEHP exposure and microbiota modifications in newborns.

Zhao et al. [93] conducted a detailed study of the intestinal microbiota composition in the different parts of the intestine after having exposed male rats to DEHP. The most notable intestinal damage occurred in the jejunum where *Mollicutes* stood out, overrepresented, and the genus *Allobaculum*, which was underrepresented. The researchers also saw that DEHP disrupted steroidogenesis in animals, thus suggesting that this compound may affect the regulation of sex hormones. Considering that it has been shown that the gut–brain axis is involved in the regulation of the synthesis of these hormones, this fact would establish a relationship between DEHP and the brain through the gut and consequently the microbiota. The study from Yu et al. carried [94] out with female rats showed that this compound promoted an imbalance of cholesterol mediated by the gut microbiota, fact that was corroborated with faecal transplantation experiments. It was also found that DEHP activated the farnesoid X receptor (FXR), which led to dysregulation of bile acid metabolism due to decreased microbial bile salt hydrolase (BSH) activity. Specifically, the genera *Acetivibrio, Clostridium, Lachnospiraceae, Lactobacillus, Ruminococcus,* and *Bacteroidales* decreased, and *Akkermansia, Desulfovibrionaceae*, and *Ocillospiraceae* augmented (detailed information in Table 3). Spearman‘s correlation analysis demonstrated the association between disrupted microbiota and metabolite alterations caused by DEHP. Considering that neuroinflammation has been related to altered metabolism of cholesterol and circulating bile acid metabolites, it is crucial to consider the role of the microbiota in this metabolic dysregulation because it ultimately implies brain damage [95]. A summary of DEHP exposure and microbiota modifications is provided in Figure 4.

#### 3.1.4. Perfluoroalkylated and Polyfluoroalkylated Substances (PFAS)

The perfluoroalkylated and polyfluoroalkylated substances, also known as PFAS, are a complex group of artificial chemical compounds that contain a fully or partially fluorinated carbon backbone linked to other functional groups. Exposure to PFAS has toxicological effects in humans, related to diseases such as cancer or diabetes, neurodegeneration, and other cardiovascular disorders [70]. The main source of exposure is the consumption of contaminated food or drinking water [96].

To understand the relationship between gut microbiota maturation and chronic exposure to certain chemical mixtures, Gardner et al. [97] characterized the microbiomes of 3- to 6-year-old children looking for correlations with semi-volatile organic compounds (SVOC) such as PFAS. They found that the concentrations of PFAS reached 10.5 ng/mL and that the dominant microbial communities were *Clostridiales, Bacteroidales, Bifidobacteroidiales,* and *Erysipelotrichales*. The populations of *Legionellales, Thermogemmatisporales,* and *Stigonematales* were strongly related to PFAS. 

It has been described that perfluorooctane sulfonate or PFOS can cause neurotoxicity and metabolic dysregulation, but few studies have evaluated its relationship with the intestinal microbiota. For this reason, Zhang et al. [98] carried out a study with male mice, where they observed that PFOS induced an increase in *Bacteroidetes* and a decrease in *Firmicutes* (lowest dose). PFOS caused the rise of *Clostridium* and *Streptococcus* and in turn, the reduction of *Flavonifractor* and *Alistipes* (details in Table 4). Bacteria belonging to the phyla *Firmicutes* were positively correlated with liver lipids, choline metabolites, branched-chain amino acids, and SCFA, while the *Clostridium XIVa* group was also positively correlated with acetate and lactate. Their results showed that PFOS affected the metabolism of the microbiota and that therefore the toxicity of it is partly mediated by the gut microbiota.

In the case of perfluorooctanoic acid (PFOA), a study carried out by Shi et al. [99] with male mice showed that exposure to it affected spatial memory and learning and caused anxiety (more information in Table 4). At the microbiota level, PFOA increased the abundance of *Bacteroidetes* and decreased *Akkermansia, Anoxybacillus, Bifidobacterium, Gemmiger, Parabacteroides,* and *Ruminococuscus*. In addition, PFOA caused a decrease in the concentrations of SCFA and the loss of the integrity of the intestinal barrier. In order to assess the neuroinflammation, they analysed inflammation mediators in the brain and found that the LPS content and the levels of TNF-α in the cortex increased after exposure. Thus, PFOA led to cognitive deficits and caused inflammation in the gut and brain, as well as dysbiosis. The group of animals that received the faecal microbiota transplantation (FMT) treatment demonstrated that it could mitigate the symptoms, and thus it was found that microbiota plays a relevant role in the neurotoxicity of PFOA. 

### 3.2. Early-Exposure and Developmental Neurotoxicity 

The microbiota begins to colonize the intestine before birth and stabilizes in it during the first 2.5 years of life. However, exposure to environmental pollutants is already done even before the conception through the mother’s lifestyle [100]. As previously mentioned, the population is constantly exposed to a wide variety of toxics found in the environment; these affect at any age, being of special importance in individuals in the reproductive stage, pregnant women, and young children. This early exposure can have serious consequences for human health in growth and development stages such as childhood, but it can also have repercussions in adult life or old age. Several studies provide evidence that dysbiosis in early life results in long-term diseases, for example, those related to the immune system [101]. Pregnant women can transfer part of their microbiota at the time of delivery or later during lactation. Through this same communication channel between mother and infant, xenobiotics or metabolites can also be transferred [102]. The foetus or newborn does not have yet the mechanisms to eliminate these substances, nor its microbiota is yet developed to fully exercise its barrier function. Epigenetic programming and maturation of the pathways that ensure survival takes place in these critical windows of the individual‘s life, which makes it important to have detailed information on the risk posed by exposure to multiple xenobiotic compounds and their effect on the growing microbial community of infants [103,104].

In the case of EDs, a recent study by Kaur et al. [105] performed in pregnant mice showed that gut microbiota and metabolome associated were changed in offspring after exposure to low-dose BPA through the mother’s diet (details in Table 5). Their results showed that *Bacteroidales* decreased in both, but *Clostridiales* and *Desulfovibrio* were elevated only in males and *Blautia* in females. Females also showed an increased number of rhamnose, deoxycholic acid, metabolites involved in carbohydrate metabolism, and synthesis, while males had alterations in lysine degradation, phenylalanine, and tyrosine metabolism and the urea cycle. Compared with human microbiota profiles, an increase in *Blautia* is characteristic of a major depressive disorder, and high levels of deoxycholic acid are present in Alzheimer‘s disease, so they are associated with cognitive deterioration. On the other hand, the urea cycle prevents high ammonia concentrations from damaging the central nervous system. Therefore, both female and male mice would suffer neurodevelopment disorders due to the toxicity of BPA but by different routes. Liao et al. [106] evaluated the protective effects of resveratrol against liver damage caused by BPA exposure on the gut–liver axis during pregnancy (Table 5). Pregnant female rats exposed and only their male offspring were studied further (to avoid errors caused by gender differences). In the offspring, the composition of their microbiota was disrupted. The abundances of *Allobaculum, Blautia, Lactobacillaceae* and *Prevotella* increased, but *Adlercreutzia* and *Oscillospira* decreased. The authors attribute this fact to the transfer of the compound through the umbilical cord and the exchange of mother–infant blood or through the consumption of breast milk during lactation. In this study, the authors demonstrated that treatment with resveratrol butyrate ester increases the population of *S24-7*, an advantageous family for the production of SCFAs and with high glycolytic activity. This indicates once again that certain bacterial populations can be of benefit to the host since they grow when the organism is in a state of recovery. On the other hand, the decrease of *Adlercreutzia* is associated with multiple sclerosis (an autoimmunity-induced neurodegenerative disease) and that of *Oscillospira* with worst health condition since its abundance is positively related to microbial diversity [107,108].

For CPF, the study by Perez-Fenandez et al. [109] with the offspring of pregnant rats showed increased cholinergic and GABAergic system hypersensitivity and stress reaction hyperactivity (in females) (more information in Table 5). The latter can be related to altered regulation of the HPA axis. It was observed that exposure to CPF caused a significant increase in the *genus Anaerobranca, Borrelia, Brevundimonas, Butyrivibrio, Candidatus Endobugula* in both sexes *Mogibacterium* and *Pelagicoccus* and also a significant decrease in *Candidatus Contubernalis Alkalaceticum, Hyphomicrobium, Nitrincola, Paracoccus, Rhizobium, and Vogesella*. Of all the named bacteria, *Anaerobranca Zarvazinii*, specifically linked to CPF and *Butyrivibrio*, are associated with butyrate production and anti-inflammatory responses. These results are consistent with the idea that low doses of CPF can affect the development of the brain and the rest of the CNS in early stages through intestinal dysbiosis. Guardia-Escote et al. [110] evaluated low CPF exposure with apoE3 and apoE4 mice pups (homozygous for the ε3 and ε4 allele). In the results, they observed that CPF decreased the relative abundance of *Streptococcus* as well as *Verrucomicrobia* (details in Table 5). Of this last phylum highlight the genus *A. Municiphila,* a mucin-degrading bacteria associated with a healthy state and negatively correlated with diabetes and obesity. CPF also promoted the increase in the levels of isovaleric acid, an SCFA which has been found in higher concentrations in stools of depression patients. Using Pearson’s correlation, isobutyric acid was related to *Coraliomargarita akajimensis*. This data suggests that CPF alters intestinal permeability and SCFA gut production, thus influencing brain levels.

Regarding DEHP, newborns are a highly vulnerable group who are often exposed to medical devices. A prospective cohort study from 2017 in newborns who received intravenous infusions (IV) showed that these neonates had higher DEHP concentrations than the control group and also different microbiota. A significant reduction in the bacterial populations of *Rothia, Bifidobacterium longum,* *Streptococcus,* and a transient increase in *Staphylococcus* was observed. Precisely, *Bifidobacterium* has been related to normal intestinal development and it is considered a probiotic [111]. 

Lei et al. [112] used an in-vitro model together with female mice of 6–8 weeks of age (mimicking human adolescence) exposed to DEHP (more information in Table 5). In the study, the microbial composition of the animals was found to be altered in a time-dependent manner. The researchers observed a greater abundance of *Lachnoclostridium*, which has been associated with neurodevelopmental disorders in human studies. MEHP (DEHP metabolite) was also detected dose-dependently in the exposed group. In the experiment with the caecal microbiota culture, the *Lactobacillus* genus was the one that decreased the most while *Parabacteroides* was the one that most increased. The production of serotonin was detected as well as metabolites of tryptophan and tyrosine derivatives. The increase in cresol related to *Lachnoclostridium boltea* and decreased butyric acid also stand out. These results showed that exposure to DEHP can directly modify the microbiota and increase the production of the metabolite cresol, a potential neurotoxin linked to behavioural abnormalities. Thus, a direct relationship is established between the neurotoxic effects of DEHP and dysbiosis.

Concerning PFAS, G. Wang, Sun, et al. [113] investigated its effects in young male mice orally exposed to PFOS (Table 5). Their results showed that PFOS caused inflammation in the liver and colon, along with impaired cholesterol and glucose metabolism. *Proteobacteria* and *Bacteroides* microbial populations increased, both changes occurred in a dose-dependent manner. In the high and medium doses, *Erysipelotrichaceae* decreased significantly, in the high and low doses, they were *Clostridial* and *Enterobacterial*, and in the three experimental groups, they were *Gammaproteobacteria* and *Blautia.* In contrast, it increased significantly in the high-dose *Ruminococcaceae* and in all groups, *Rikenellaceae*. The production of SCFA (especially acetic acid) was also reduced in the medium and high doses, together with the expression of occludin (tight junction protein). The biosynthesis of steroid and flavonoid hormones was disturbed. Therefore, this study showed that PFOS damage both the environment and the intestinal barrier, modifying the microbiota and its products. As discussed earlier in Section 2, this toxicity in the liver and colon may be the cause of brain damage.

**Figure 5:**
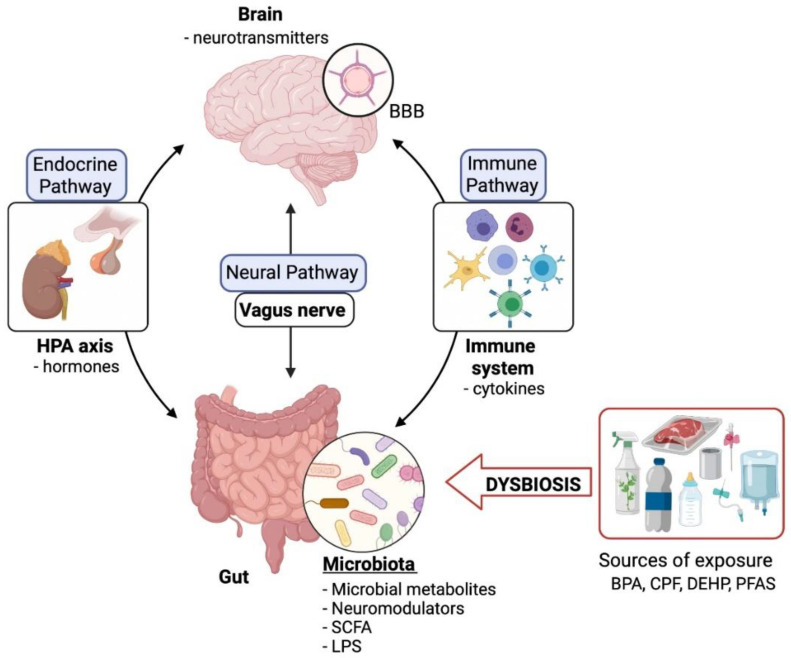
Impact of contaminants on the microbiota–gut–brain axis and linkage with neurotoxicity.

On the other hand, a recent study carried out by G. Wang et al. [114] with subacute and subchronic exposure to PFOA showed that it caused significant changes in the abundance of the intestinal microbiota of juvenile mice that partly promoted liver inflammation and oxidative stress (more details in Table 5). Exposures to PFOA caused an imbalance of inflammatory cytokines, induced stress, and promoted liver disease, in addition to altering the intestinal environment and causing changes in the composition of the microbiota. A decrease was seen in the *Lactobacillus, Bifidobacterium,* and *Dehalobacterium* genera. In the subacute groups, the *Porphyromonadaceae* and *Parabacteroides* increased, while in the sub-chronic groups, a reduction was induced in the ratio of *Firmicutes* to *Bacteroidetes*. SCFAs (especially butyric acid) were also significantly reduced. As a result, it was confirmed that the damage induced by PFOA interacted with the intestinal microbiota since the proportion of probiotics was reduced and the microbiota populations associated with liver injury increased. A summary of possible impacts of contaminants on the microbiota–gut–brain axis and linkage with neurotoxicity is presented in Figure 5.

**Table 5:** Studies on early exposure to BPA, CPF, DEHP, and PFAS and their association with the intestinal microbiota.

BPA Exposure						
Cell Line/Species of Animal	Dose	Duration	Bacterial Genus	Bacterial Phylum	Impact *	Reference
Male and female offspring of California mice dams	5 mg/kg feed weight (LD) and 50 mg/kg feed weight (UD) (administered to dams)	Two weeks prior to breeding, throughout gestation, and lactation.	*Blautia*	*Firmicutes*		[105]
Female offspring			*Desulfovibrio*	*Proteobacteria*	↑	
Male offspring				*Firmicutes* (*Clostridiales*)	↑	
				*Bacteroidetes* (*Bacteroidales*)	↓	
Male offspring of pregnant females Sprague Dawley rats	50 μg/kg/day (administered to dams)	30 days of gestation and 21 days of lactation	*Blautia*	*Firmicutes*	↑	[106]
			*Prevotella*	*Bacteroidetes*	↑	
			*Allobaculum*	*Firmicutes*	↑	
				*Firmicutes* (*lactobacillaceae*)	↑	
			*Adlercreutzia*	*Actinobacteria*	↓	
			*Oscillospira*	*Firmicutes*	↓	
**CPF Exposure**						
**Cell Line/Species of Animal**	**Dose**	**Duration**	**Bacterial Genus**	**Bacterial Phylum**	Impact *	**Reference**
Male and female offspring of pregnant female Wistar rats	1 mg/kg/mL/day	Six days (PND10-PND15)	*Anaerobranca*	*Firmicutes*	↑	[109]
			*Borrelia*	*Spirochaetes*	↑	
			*Brevundimonas*	*Proteobacteria*	↑	
			*Butyrivibrio*	*Firmicutes*	↑	
			*Candidatus* *Endobugula*	*Firmicutes*	↑	
			*Mogibacterium*	*Firmicutes*	↑	
			*Pelagicoccus*	*Verrucomicrobia*	↑	
			*Candidatus* *Contubernalis*	*Firmicutes*	↓	
			*Hyphomicrobium*	*Proteobacteria*	↓	
			*Nitrincola*	*Proteobacteria*	↓	
			*Paracoccus*	*Proteobacteria*	↓	
			*Rhizobium*	*Proteobacteria*	↓	
			*Vogesella*	*Proteobacteria*	↓	
Male C57BL/6, apoE3 and apoE4 mice (homozygous for the ε3 and ε4 allele)	1 mg/kg/mL/day	Six days (PND10-PND15)	*Streptococcus*	*Firmicutes*	↓	[110]
			*Akkermansia*	*Verrucomicrobia*	↓	
			*Luteolibacter*	*Verrucomicrobia*	↓	
			*Prosthecobacter*	*Verrucomicrobia*	↓	
			*Coraliomargarita*	*Verrucomicrobia*	↓	
**DEHP Exposure**						
**Cell Line/Species of Animal**	**Dose**	**Duration**	**Bacterial Genus**	**Bacterial Phylum**	Impact *	**Reference**
Female C57BL/6 mice	1 and 10 mg/kg body weight/day	14 days	*Lachnoclostridium*	*Firmicutes*	↑	[112]
			*Akkermansia*	*Verrucomicrobia*	↓	
			*Odoribacter*	*Bacteroidetes*	↓	
			*Clostridium*	*Firmicutes*	↓	
Anaerobic culture of caecal microbiota	10 and 100 μM	Seven days		*Parabacteroidetes*	↑	
			*Lactobacilllus*	*Firmicutes*	↓	
				*Firmicutes*	↓	
**PFAS Exposure**						
**PFOS**						
**Cell Line/Species of Animal**	**Dose**	**Duration**	**Bacterial Genus**	**Bacterial Phylum**	Impact *	**Reference**
Male C57BL/6J mice	0.3, 3 and 30 μg/g BW/day	16 days		*Bacteroidetes* (*Rikenellaceae*)	↑	[113]
				*Proteobacteria* (*Gammaproteobacteria*)	↓	
			*Blautia*	*Firmicutes*	↓	
**PFOA**						
**Cell Line/Species of Animal**	**Dose**	**Duration**	**Bacterial Genus**	**Bacterial Phylum**	Impact *	**Reference**
Juvenile C57BL/6J mice	3 and 30 mg/kg BW (Subacute exposure)	14 days		*Bacteroidetes (Porphyromonadaceae*)	↑	[114]
			*Parabacteroides*	*Bacteroidetes*	↑	
	2.5, 5 and 10 mg/kg BW (Subchronic exposure)	30 days		*Bacteroidetes*	↑	
				*Firmicutes*	↓	
	Both doses	Respective days	*Lactobacilllus*	*Firmicutes*	↓	
			*Bifidobacterium*	*Actinobacteria*	↓	
			*Dehalobacterium*	*Firmicutes*	↓	

Impact *: In this column, the increase (↑) or decrease (↓) refers to bacterial populations depending on the effect of the toxic.

## 4. Existing Gaps in the Microbiota Research

The study of microbiota has grown in recent years, leading to improved knowledge about microbiota relationship with the human body, especially the brain. The MGBA plays a crucial role in individual‘s health, specifically to avoid neurological disorders. Hence, it is important to understand the mechanisms for maintaining this homeostasis. In-vitro, in-vivo, and in-silico studies have allowed significant advances in understanding these microorganisms and their connection with diseases and toxicity. However, we still have some gaps to resolve the complexity of the interplay between the MGB axis and to use microbiota as a promising therapeutic approach to reduce neurological diseases.

One of these gaps is that most studies are performed with animals, but there are differences in the microbiota profile between humans and animals. Although a pattern is maintained, the proportion of the bacteria phylum is not the same in humans as in mice. Nagpal et al. [115] showed (from faecal samples from the same period) that the human gut was dominated by *Bacteroides* (27.5% of relative abundance), *Ruminococcaceae* (10.2%), and *Clostridiales* (9.7%) while in the mouse gut were the *family S24-7* (44.7%) and *Clostridiales* (25.3%) predominating. In the study by Pan et al. [116], it was also observed that the percentage of *Ruminococcus* genes was higher in humans than in mice, while the percentage of *Clostridium* genes was higher in mice than in humans. 

Another issue is that a single compound is studied in most experimental studies instead of a combination of xenobiotics, which is more plausible with everyday exposure. Genetics and environmental factors such as lifestyle play a role in determining microbiota profile; that is why, in addition to studying microbial populations, we must establish microbial biomarkers that we can relate to specific functions, metabolic mechanisms or disorders [117]. Among the potential biomarkers, one are SCFAs, used for studying diseases like colorectal cancer and Parkinson‘s disease [118,119]. Apart from bacteria, fungi and viruses are also part of microbiota which have not been studied in so much detail.

Regarding the models used to study the microbiota–xenobiotic relationship, animal experiments are the most commonly used. It is true that animal experimentation is essential to understand the toxicological impact of compounds in-vivo, but these often require a lot of time, money and involve dealing with ethics. On the other hand, in-silico simulations and new approaches derived from bioengineering such as organs-on-chip (OoCs) or 3D cultures can minimize experiments and save time. These advanced techniques will help refine and reduce the number of animals used and step towards promoting the 3 Rs (Replace, reduce, and refine) principle. 

Lastly, the challenge is to understand the molecular mechanisms that regulate the microbiota and perform the computational analysis with the enormous amount of data generated by the meta-omics. To understand the relationship between microbiota and neurotoxicity, it is crucial to determine the causality between the impact of the xenobiotic on the microbiota and that on the brain. 

## 5. In-Silico Models for Microbiota-Chemical Interaction and Future Prospects

As discussed previously, the intestinal microbiota is a complex biological system including many elements that interact with each other and the host, giving rise to intertwined feedback loops. To better understand this system and to be able to predict it, researchers have worked on metagenomic modelling. NGS and metagenomics are used to determine the set of species or genes of the microbiota. Through computational tools, it has been possible to define the composition of a healthy microbiota [120] and identify significant associations between the microbiota of healthy and sick people [121], but challenges still lie in determining the mechanistic connection of how dysbiosis causes or promotes diseases and the role environmental pollutants play in it [122]. 

Flux balance analysis (FBA) is a kind of constraint-based reconstruction and analysis (COBRA) method often used, which consists of a reconstruction of a metabolic network where the metabolites are the nodes of the network [123]. One of the uses of these metabolic networks is to predict the effect of alterations in microbiota. In order to use FBA, the metabolic capacity of the bacteria that make up the microbiota network must be known, which can be done by genomic-scale metabolic models (GEM) or genomic-scale metabolic reconstructions (GENRE). These metabolic networks can be extrapolated to include those of the host, which makes it possible to predict the effect of the microbiota on host health. Other in-silico models are OptCom (Python), MICOM (Python), BacArena (Python and R), COMETS (Python and MATLAB), FLYCOP (Python), MiMoSa (Python), and Recon3D (R and MATLAB) [124].

Nowadays, research is moving towards developing physiologically-based pharmacokinetic models (PBPK) for xenobiotics and their metabolites, incorporating microbiota-induced metabolic activity. Such kinds of models provide insights into how the gut microbiome can influence chemical pharmacokinetics. For instance, Kogadeeva et al. developed a PBPK model for three different drugs to quantify the microbiota role in the metabolism of xenobiotics and predict different parameters related to host and microbiota [125]. A similar approach can be utilized to develop a PBPK model for environmental chemicals and further can be combined with systems biology models which include FBA to predict the effect from exposure, chemical pharmacokinetics, the role of microbiota, and adverse effects on the host [126]. However, research on integrated models is still limited due to data scarcity and limited understanding of steady-state assumptions in models like COBRA and molecular details of PBPK Models. 

Modelling the intestinal microbiota is also challenging because a dynamic spatial organization must be taken into account since bacteria can move along the intestinal tract, along with pH gradients and variable concentrations of metabolites, nutrients, or oxygen. The behaviour and metabolic capacity of the strains in the environment to be investigated must also be known, which makes it difficult to model new species that are difficult to cultivate in the laboratory and therefore little studied [127,128]. In addition, these models are mathematical descriptions of what happens in reality, and they need to be validated experimentally to show that they accurately represent the physiology of the real system. To provide the experimental data, there are innovative projects with OoCs or microphysiological systems that aim to study the interactions between the microbiota and the host [129,130].

## 6. Conclusions

The microbiota has a relevant role in the neuro–immune–endocrine connection, and there is sufficient evidence that xenobiotics interact with it. Although there is already research about its composition, there is no clear consensus on the species profile that is altered by xenobiotics in each case. This may be due to the fact that within the same bacterial pylum, there are genera that react differently to the same compound. In order to fully explain this, more studies are needed that delve into this topic. This review involve thorough literature search for demonstrating the relationship between neurotoxicity and the microbiota. Based on this, we consider that the microbiota should be included as a parameter for decision-making by regulatory agencies, since it conditions the vulnerable population and hence human health.

Finally, to emphasize that the new 3D in-vitro models and the recent advances in in-silico tools open a wide range of possibilities to understand these intestinal microorganisms and thus avoid their dysbiosis. 

## Figures and Tables

**Table 3:** Studies on exposure to DEHP and its relationship with the intestinal microbiota.

DEHP Exposure						
Cell Line/Species of Animal	Dose	Duration	Bacterial Genus	Bacterial Phylum	Impact *	Reference
Female ICR mice	500 and 1500 mg/kg bw/day	30 days	*Turicibacter*	*Firmicutes*	↑	[91]
			*Akkermansia*	*Verrucomicrobia*	↑	
			*Romboutsia*	*Firmicutes*	↑	
				*Bacteroidetes*	↓	
				*Epsilonbacteraeota*	↓	
				*Actinobacteria*	↓	
Male SD rats	0, 300, 1000 and 3000 mg/kg bw/day	30 days		*Proteobacteria*	↑	[92]
			*Mycoplasma*	*Tenericutes*	↑	
			*Actinomyces*	*Actinobacteria*	↑	
			*Porphyromonas*	*Bacteroidetes*	↑	
			*Peptostreptococcaceae*	*Firmicutes*	↑	
			*Sutterella*	*Proteobacteria*	↑	
Male SD rats	500 mg/kg bw/day	14 days		*Tenericutes* (*Mollicutes*)	↑	[93]
			*Allobaculum*	*Firmicutes*	↓	
Female SD rats	0.5 mg/kg bw/day	23 weeks	*Akkermansia*	*Verrucomicrobia*	↑	[94]
			*Oscillibacter*	*Firmicutes*	↑	
			*Pseudoflavonifractor*	*Firmicutes*	↑	
				*Proteobacteria* (*Desulfovibrionaceae*)	↑	
				*Firmicutes* (*Ruminococcaceae*)	↑	
			*Acetivibrio*	*Firmicutes*	↓	
			*Alloprevotella*	*Bacteroidetes*	↓	
			*Barnesiella*	*Bacteroidetes*	↓	
			*Clostridium*	*Firmicutes*	↓	
				*Firmicutes* (*Lachnospiraceae*)	↓	
			*Lactobacilllus*	*Firmicutes*	↓	
			*Prevotella*	*Bacteroidetes*	↓	
			*Roseburia*	*Firmicutes*	↓	
			*Ruminococcus*	*Firmicutes*	↓	
				*Bacteroidetes* (*Porphyromonadaceae*)	↓	

Impact *: In this column, the increase (↑) or decrease (↓) refers to bacterial populations depending on the effect of the toxicity.

**Table 4:** Studies on exposure to PFAS and its relationship with the intestinal microbiota.

PFAS Exposure						
**PFOS**						
**Cell Line/Species of Animal**	**Dose**	**Duration**	**Bacterial Genus**	**Bacterial Phylum**	Impact *	**Reference**
Male C57BL/6J mice	0, 0.003%, 0.006%, and 0.012%	3 weeks	*Clostridium*	*Firmicutes*	↑	[98]
			*Streptococcus*	*Firmicutes*	↑	
				*Bacteroidetes*	↑	
			*Flavonifractor*	*Firmicutes*	↓	
			*Alistipes*	*Bacteroidetes*	↓	
**PFOA**						
**Cell Line/Species of Animal**	**Dose**	**Duration**	**Bacterial Genus**	**Bacterial Phylum**	Impact *	**Reference**
Male C57BL/6J mice	0, 0.5, 1, and 3 mg/kg (bw)/day	35 days		*Bacteroidetes*	↑	[99]
			*Akkermansia*	*Verrucomicrobia*	↓	
			*Bifidobacterium*	*Actinobacteria*	↓	
			*Ruminococcus*	*Firmicutes*	↓	
			*Anoxybacillus*	*Firmicutes*	↓	
			*Gemmiger*	*Firmicutes*	↓	
			*Parabacteroides*	*Bacteroidetes*	↓	

Impact *: In this column, the increase (↑) or decrease (↓) refers to bacterial populations depending on the effect of the toxicity.

## Data Availability

Not applicable.

## Abbreviations

BPA: Bisphenol A; CPF: Chlorpyrifos; DEHP: Diethylhexyl phthalate; PFAs: Per- and polyfluoroalkyl substances; MGBA: microbiota-gut-brain axis; BBB: Blood-brain barrier; CNS: Central nervous system; ANS: Autonomic nervous system; ENS: enteric nervous system; SNS: Sympathetic nervous system; PNS: Parasympathetic nervous system; SCFA: Short-chain fatty acids; EEC: enteroendocrine cells; TLR: Toll-like receptors; LPS: Lipopolysaccharide; GABA: y-aminobutyric acid; Ach: Acetylcholine; HDAC: histone deacetylase; HPA: Hypothalamic-pituitary-adrenal axis; NPY: Neuropeptide Y; GLP: Glucagon-like peptide; MAMPs: Microbial-associated molecular patterns; TNF-α: Tumor necrosis factor alpha; IL: Interleukin; CRH: Corticotropin-releasing hormone; AVP: Arginine vasopressin; ED: Endocrine disruptor; EDCs: endocrine-disrupting chemicals; SHIME: Simulator of the human intestinal microbial ecosystem; NGS: next-generation sequencing; TDI: Daily intake tolerable; HFD: High fat diet; HSD: High sucrose diet; 5-HT: 5-hydroxytryptamine; TRP: tryptophan; 5-HIAA: 5-hydroxyindoleacetic acid; AChE: Acetylcholinesterase; EFSA: European Food Safety Authority; MAIT: mucosal-associated invariant; PYY: Peptide tyrosine tyrosine; AA: amino acid; FXR: farnesoid X receptor; BSH: microbial bile salt hydrolase;; PSD: postsynaptic density; SAP-95: synapse-associated protein 95; FMT: Fecal microbiota transplantation; OoCs: organs-on-chip; FBA: flux balance analysis; COBRA: constraint-based reconstruction and analysis; GEM: genomic-scale metabolic models; GENRE: genomic-scale metabolic reconstructions.
